# Successful use of dupilumab for egg-induced eosinophilic gastroenteritis with duodenal ulcer: a pediatric case report and review of literature

**DOI:** 10.1186/s13223-023-00859-3

**Published:** 2023-12-05

**Authors:** Mitsuru Tsuge, Kenji Shigehara, Kazuhiro Uda, Seiji Kawano, Masaya Iwamuro, Yukie Saito, Masato Yashiro, Masanori Ikeda, Hirokazu Tsukahara

**Affiliations:** 1 Department of Pediatric Acute Diseases, Okayama University Academic Field of Medicine, Dentistry, and Pharmaceutical Sciences, 2-5-1 Shikata-cho, Kita-ku, Okayama, 700-8558 Japan; 2 Department of Pediatrics, Okayama University Hospital, Okayama, Japan; 3 Department of Gastroenterology and Hepatology, Okayama University Hospital, Okayama, Japan; 4 Department of Pediatrics, Okayama University Academic Field of Medicine, Dentistry, and Pharmaceutical Sciences, Okayama, Japan

**Keywords:** Duodenal ulcer, Dupilumab, Eosinophilic gastroenteritis, Eotaxin-3, Food allergy, Interleukin-5, Interleukin-13, Non-esophageal eosinophilic gastrointestinal disorder

## Abstract

**Background:**

Non-esophageal eosinophilic gastrointestinal disorder (non-EoE-EGID) is a rare disease in which eosinophils infiltrate parts of the gastrointestinal tract other than the esophagus; however, the number of patients with non-EoE-EGID has been increasing in recent years. Owing to its chronic course with repeated relapses, it can lead to developmental delays due to malnutrition, especially in pediatric patients. No established treatment exists for non-EoE-EGID, necessitating long-term systemic corticosteroid administration. Although the efficacy of dupilumab, an anti-IL-4/13 receptor monoclonal antibody, for eosinophilic esophagitis, has been reported, only few reports have demonstrated its efficacy in non-EoE EGIDs.

**Case presentation:**

A 13-year-old boy developed non-EoE-EGID with duodenal ulcers, with chicken eggs as the trigger. He was successfully treated with an egg-free diet, proton pump inhibitors, and leukotriene receptor antagonists. However, at age 15, he developed worsening upper abdominal pain and difficulty eating. Blood analysis revealed eosinophilia; elevated erythrocyte sedimentation rate; and elevated levels of C-reactive protein, total immunoglobulin E, and thymic and activation-regulated chemokines. Upper gastrointestinal endoscopy revealed a duodenal ulcer with marked mucosal eosinophilic infiltration. Gastrointestinal symptoms persisted even after starting systemic steroids, making it difficult to reduce the steroid dose. Subcutaneous injection of dupilumab was initiated because of comorbid atopic dermatitis exacerbation. After 3 months, the gastrointestinal symptoms disappeared, and after 5 months, the duodenal ulcer disappeared and the eosinophil count decreased in the mucosa. Six months later, systemic steroids were discontinued, and the duodenal ulcer remained recurrence-free. The egg challenge test result was negative; therefore, the egg-free diet was discontinued. Blood eosinophil count and serum IL-5, IL-13, and eotaxin-3 levels decreased after dupilumab treatment. The serum levels of IL-5 and eotaxin-3 remained within normal ranges, although the blood eosinophil counts increased again after discontinuation of oral prednisolone.

**Conclusions:**

Suppression of IL-4R/IL-13R-mediated signaling by dupilumab may improve abdominal symptoms and endoscopic and histologic findings in patients with non-EoE-EGID, leading to the discontinuation of systemic steroid administration and tolerance of causative foods.

**Supplementary Information:**

The online version contains supplementary material available at 10.1186/s13223-023-00859-3.

## Background

Eosinophilic gastrointestinal disorders (EGIDs) are chronic inflammatory diseases caused by extensive eosinophilic infiltration in the gastrointestinal tract. EGIDs are divided into eosinophilic esophagitis (EoE), in which eosinophils infiltrate only the esophagus, and non-esophageal eosinophilic gastrointestinal disorder (non-EoE-EGID), in which eosinophils infiltrate the digestive tract other than the esophagus [1]; both EoE and non-EoE-EGID can be associated. Non-EoE-EGID is rare with a prevalence of 1–20 cases per 100,000 people and less common than EoE [2]; however, reports on non-EoE-EGID have been increasing in recent years [3].

Non-EoE-EGID causes various gastrointestinal symptoms, such as anorexia, abdominal pain, vomiting, diarrhea, gastrointestinal bleeding, and ascites. Since patients with non-EoE-EGID have a chronic course with repeated relapses of abdominal symptoms [4], it leads to malnutrition and worsens the quality of life [5]; therefore, early induction and long-term maintenance of remission are important for developmental prognosis, particularly in pediatric patients.

Atopic disorders are frequent comorbidities in patients with EGIDs, and a recent systematic review with meta‐analysis reported that the prevalence of atopic comorbidities ranged from 25 to 54% in patients with non-EoE-EGIDs [6]. However, there are currently no established pharmacological treatments for non-EoE-EGIDs. Various treatments, such as systemic steroids, leukotriene receptor antagonists (LTRAs), antihistamines, and food elimination therapy are administered; however, relapse often occurs even with these treatments [7]. Dupilumab, an anti-interleukin (IL)-4/13 receptor monoclonal antibody, has been used to treat atopic diseases such as severe asthma and moderate-to-severe atopic dermatitis. In recent years, the efficacy of dupilumab in EoE has been reported [8], and it is also expected to be effective in non-EoE EGIDs [9]. However, only a few case reports have demonstrated its efficacy in non-EoE EGID, and changes in serum biomarkers have not yet been reported.

Herein, we report a pediatric case of egg-induced non-EoE-EGID with a duodenal ulcer, in which dupilumab administration led to ulcer healing, disappearance of eosinophilic infiltration, discontinuation of systemic steroids, and acquisition of egg tolerance. In addition, we reported changes in serum Th2 cytokines and eotaxin-3 levels following dupilumab administration.

## Case presentation

A 13-year-old boy visited our hospital with persistent epigastric and right upper quadrant pain, loss of appetite, and nausea, and upper gastrointestinal endoscopy revealed ulcers extending from the gastric antrum to the duodenum and marked mucosal eosinophilic infiltration (288 /high-power field [HPF]). *Helicobacter pylori* was not detected in the mucosal tissue or stool, and serum *Helicobacter pylori*-specific IgG antibody testing showed negative results. The patient had no history of non-steroidal anti-inflammatory drug (NSAID) intake. He had comorbidities, including atopic dermatitis and allergic rhinitis, but had no family history of atopic diseases. At the time of the hospital visit, atopic dermatitis and allergic rhinitis were mild and well-controlled with topical and nasal steroids. His abdominal symptoms and duodenal ulcer improved with a six-food elimination therapy (eggs, milk, wheat, soybeans, seafood, and nuts), LTRA (montelukast), proton pump inhibitors (PPI, esomeprazole), and intravenous systemic steroids (prednisolone: 1.5 mg/kg/day, 60 mg/day). Based on the Japanese clinical practice guidelines for eosinophilic gastrointestinal disease in infants and adults, LTRA was administered orally. He developed steroid-induced glaucoma during systemic steroid administration. Oral steroids were thereafter tapered off for 2 months, and the decrease in mucosal eosinophilia and shrinkage of ulcers were confirmed by gastroscopy. A food challenge test was carried out by adding one of the six types of food, which was previously eliminated, every week (see Additional file 1: Fig. S1). As a result, on the 4th day after starting an egg diet, a clear recurrence of epigastralgia was observed, and the abdominal symptoms disappeared after the egg-free diet was resumed. For confirmation, the cancellation of egg elimination from the diet as described above was performed twice, but similar results were obtained. On the other hand, other foods have been successfully reintroduced. We did not perform additional gastroscopies and histopathological examinations and confirmed eggs as one of the possible causative allergens based on the abdominal symptoms. The lymphocyte stimulation test (LST) was performed at an external facility (Bio Medical Laboratories, Saitama, Japan) to quantify the extent of lymphocyte proliferation by culturing the whole blood of the patient with chicken egg, and the result was positive (2.5 times compared with no stimulation, reference value: < 1.8 times). The patient continued on an egg-free diet, PPI, and LTRA, and his gastrointestinal symptoms disappeared and remained in remission without recurrence for 3 months.

However, at the age of 15 years, he was referred to our hospital because he had upper abdominal pain again. At the time of consultation, epigastric and right upper quadrant tenderness was observed without muscle defense or rebound tenderness. He presented with itchy erythema, lichenification, and desquamation of the forehead, cheeks, scalp, neck, cubital fossa, and popliteal fossa, and was diagnosed with eczema. He had no history of NSAID intake at this time. Blood analysis showed normal white blood cell count (6010/µL), eosinophilia (1747/μL), thrombocytosis (43.2 × 10^4^/μL), elevated erythrocyte sedimentation rate (28 mm/h), and mild anemia (hemoglobin 10.5 g/dL). Decreased serum iron (11 μg/dL) and ferritin (13.9 ng/mL) levels and elevated total iron-binding capacity (440 μg/dL), suggestive of iron deficiency. No abnormal blood cell morphologies or atypical cells were observed in the peripheral blood. No coagulopathy, hypoproteinemia, hepatic dysfunction, renal hypofunction, or electrolyte abnormalities were observed. High serum levels of C-reactive protein (CRP) (1.31 mg/dL), total immunoglobulin E (IgE) (768 IU/mL), and thymus and activation-regulated chemokine (TARC) (1030 pg/mL) were observed. Stool culture, parasite tests, and serum autoantibody were negative. No hypocomplementemia or hyperglobulinemia was observed. Bone marrow biopsy was not performed; therefore, malignancies such as myeloproliferative disease, chronic eosinophilic leukemia, and acute leukemia could not be completely ruled out. Computed tomography revealed no intestinal wall thickening or neoplastic lesions. *Helicobacter pylori* antigen was not detected in stool. Upper gastrointestinal endoscopy revealed a well-demarcated punch-like duodenal ulcer with surrounding edematous thickening. Pathological findings of a mucosal biopsy of the ulcer site showed marked eosinophilic infiltration in the mucosal tissue centered on the lamina propria (96/HPF) with no evidence of inflammatory bowel disease, malignant lymphoma, or granulomatous vasculitis.

Thereafter, oral prednisolone (0.7 mg/kg/day, 30 mg/day) was started while the egg-free diet, PPI, and LTRA were continued since the initial diagnosis of the ulcer. However, epigastralgia and feeding difficulties persisted even after treatment initiation, making it difficult to reduce the oral steroid dose. Moreover, despite the daily use of potent-class topical steroids for exacerbated atopic dermatitis, no improvement in the dermatitis was observed for months. The systemic Eczema Area and Severity Index [EASI] and head-neck EASI scores were 15.1 and 3.2, respectively. Dupilumab was initiated because strong topical steroids were not effective and the eruptions accompanied by strong inflammation remained over a wide area; treatment with dupilumab was initiated with patient and parental consent (600 mg initially and 300 mg every 2 weeks thereafter).

**Fig. 1:**
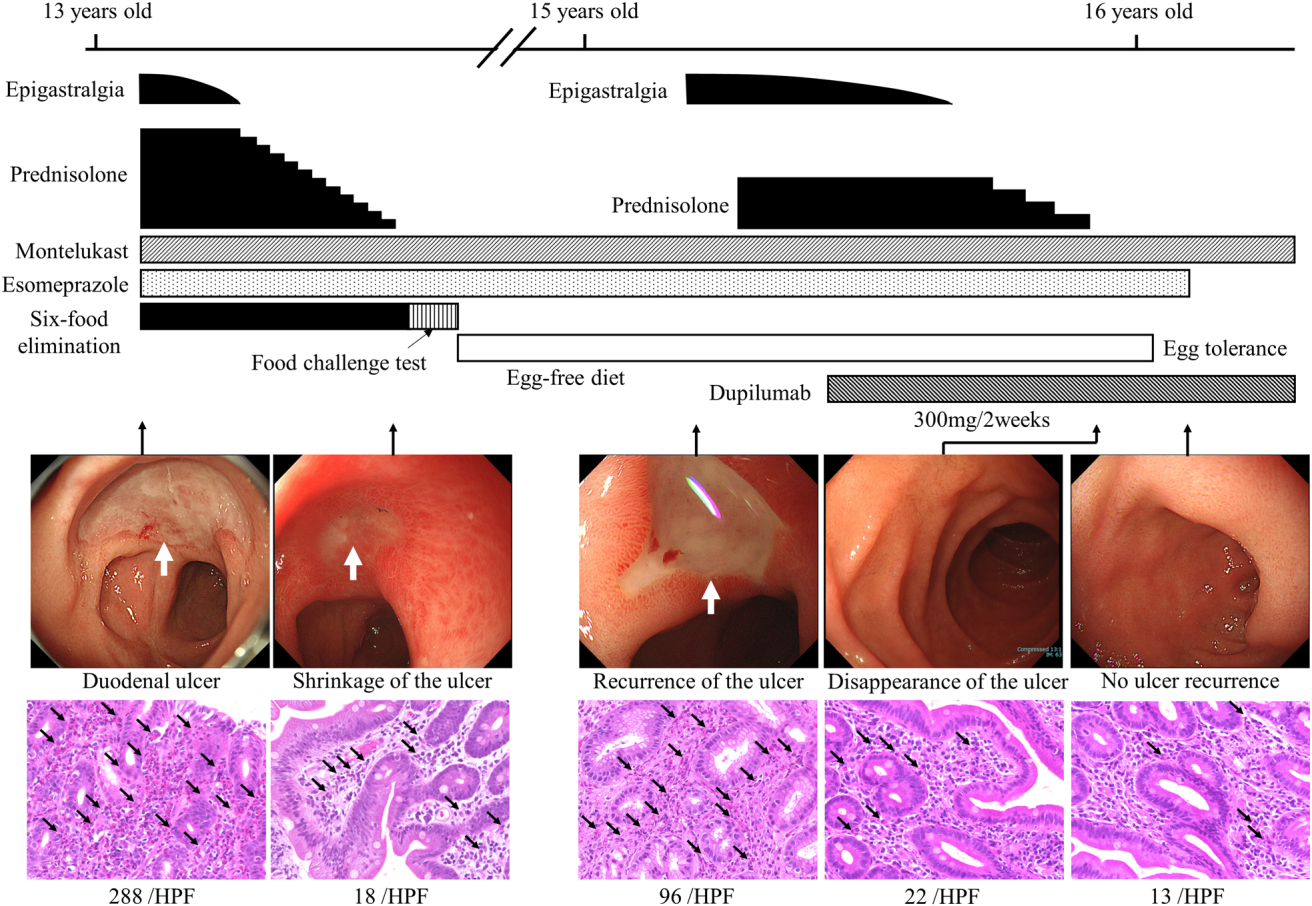
The clinical course of abdominal symptoms, treatment interventions, gastroscopy findings, and pathological findings of biopsy tissue. The upper photographs show the gastrointestinal endoscopic findings of duodenal ulcer, and the lower photographs show the pathological findings of the biopsied mucosa (hematoxylin and eosin stain [H&E] × 400), along with the number of infiltrated eosinophils (cells/high-power field [HPF]). White arrows indicate duodenal ulcers. Black arrows indicate eosinophils in the mucosa

Approximately 3 months after starting dupilumab, the epigastralgia subsided and the oral prednisolone dose was reduced to 15 mg/day. Upper gastrointestinal endoscopy performed 5 months after starting dupilumab showed the disappearance of the duodenal ulcer and a decreased eosinophil count in the mucosa (22/HPF) (Fig. 1). The prednisolone dose was tapered and discontinued after 6 months of dupilumab administration. During this period, no recurrence of abdominal symptoms was observed, and upper gastrointestinal endoscopy performed 2 months after prednisolone discontinuation showed healing of the duodenal ulcer and eosinophil infiltration within the normal range (13/HPF). Additionally, the severity of atopic dermatitis also improved to a systemic EASI score of 1.6 and a head-neck EASI score of 0.2. No recurrence of abdominal symptoms was observed after stopping the egg-free diet for 2 weeks, and the egg-free diet was discontinued thereafter. Blood eosinophil counts, total IgE levels, and TARC levels remained elevated even after oral prednisolone initiation but decreased after dupilumab initiation (Table 1). The duodenal ulcer remained recurrence-free for at least 6 months. Re-examination for egg-specific LST against chicken eggs was negative at 16 months after starting dupilumab. We measured serum levels of IL-5, IL-13, and eotaxin-3 using an enzyme-linked immunosorbent assay (R&D Systems, Minneapolis, MN, USA), and these levels also did not decrease even after oral prednisolone initiation but declined after dupilumab initiation. Although the blood eosinophil count increased again after discontinuation of prednisolone, serum levels of IL-5 and eotaxin-3 remained within normal ranges. We also examined changes in fecal calprotectin levels over time; however, the levels increased after starting dupilumab administration, with no significant changes associated with the improvement in clinical symptoms.

**Table 1:** Changes in blood/fecal biomarkers associated with allergic/eosinophilic inflammation during dupilumab treatment

Duration from the recurrence of duodenal ulcer (months)	0	3	6	9	12	15	18	20
	PSL Start			DUP Start		PSL Off		Egg tolerance
Eosinophil counts (/µl)(NR: 70—450)	1747	747	365	598	126	366	412	461
Total IgE (U/ml) (NR: < 170)	768	ND	ND	2324	1176	582	411	368
TARC (pg/ml) (NR: < 742)	1030	ND	ND	620	237	175	129	241
IL-5 (pg/ml) (NR: < 4.0)	9.4	5.4	4.5	4.2	0.9	1.4	1.3	0.8
IL-13 (pg/ml) (NR: < 13.2)	22.4	24.4	24.3	60.4	13.7	16.4	21.7	15.1
Eotaxin-3 (pg/ml) (NR: < 16.7)	37.7	29.6	21.4	26.6	13.1	16.9	16.3	15.0
Fecal Calprotectin (mg/kg) (NR: < 50)	347	99	381	28	92	180	ND	ND

*NR* normal range, *PSL* prednisolone, *DUP* dupilumab, *IgE* immunoglobulin E, *TARC* thymus and activation-regulated chemokine, *IL* interleukin, *ND* no data

## Discussion and conclusions

Most of the reported cases of non-EoE-EGID with duodenal ulcers were of male teenagers [10–15], and half had a history of atopic complications, such as asthma or allergic rhinitis, similar to our case. A previous study reported that 7.5% of pediatric gastrointestinal ulcers were non-EoE-EGIDs [16]. Non-EoE-EGID cases with duodenal ulcers are often refractory, steroid-resistant, and recurrent [11, 12, 14].

Food is a causative trigger of EGIDs; however, identifying food antigens is often difficult because EGIDs are mainly caused by non-IgE-dependent food allergies. Antigen-specific LST reveals the involvement of non-IgE-independent cell-mediated immune response, and the antigen-specific LST was reported to be positive in a case report of non-EoE-EGID due to chicken eggs [17], as in our patient. However, we were unable to prove that the positive LST result was the cause of non-EoE EGID because atopic patients retain a variety of allergen-specific T-cell responses. Egg patch testing was not performed on this patient. Moreover, LSTs for other foods such as milk, wheat, and soy were not performed on this patient. Although reports on the efficacy of food elimination therapy exist in patients with non-EoE-EGID [18, 19], well-designed high-quality studies are lacking [19]. Our patient showed inadequate remission on an egg-free diet alone and required appropriate pharmacological treatment. Considering that recurrence was observed even after starting the egg-free diet, it was suggested that multiple unknown causative substances may be involved. Systemic steroids are often administered to induce remission in patients with non-EoE-EGID. These steroids can improve symptoms in a short period and have a high remission rate; however, relapses often occur after discontinuation. In a previous long-term cohort study, 95% of patients treated with oral corticosteroids had remission, but 58% of patients subsequently had multiple relapses and a chronic course [4]. Therefore, long-term administration is often necessary and can lead to side effects such as osteoporosis, susceptibility to infections, and glucose intolerance. Considering that our patient had a history of steroid-induced glaucoma, it was necessary to avoid long-term high-dose steroid administration. Careful steroid dose reduction was attempted; however, it could not be discontinued completely because of persistent gastrointestinal symptoms. A retrospective study of pediatric patients with non-EoE-EGID reported that topical budesonide therapy was as effective as systemic steroid administration [20]. Given that our patient's eosinophilic inflammation occurred in the stomach and duodenum, we speculated that oral administration of an inhaled budesonide suspension, which is approved for bronchial asthma as a topical steroid therapy, would be pharmacokinetically effective. We did not select it to treat our patient, because the patient and his parents did not fully agree with the efficacy of topical steroid treatment and strongly desired an early remission of severe atopic dermatitis. Therefore, dupilumab was selected as a treatment for atopic dermatitis after taking informed consent.

The pathogenesis of EGIDs, which are non-IgE-mediated food allergies, involves type 2 inflammation caused by antigen-specific Th2 lymphocytes and antigen-nonspecific innate lymphocyte type 2 (ILC2) [21, 22]. Causative agents such as food that enter the lamina propria of the intestinal tract are captured by the dendritic cells and are presented to the CD4-positive naive T cells. Cell-mediated immunity is induced by persistent stimulation, differentiation of naive T cells to type 2 helper T cells is promoted through IL-4 production, and cytokines, such as IL-4, IL-5, and IL-13, are produced by Th2 lymphocytes. Additionally, innate immune mediators such as thymic stromal lymphopoietin (TSLP) are produced by intestinal mucosal epithelium damage, and IL-5 and IL-13 are produced by ILC2. IL-5 is involved in eosinophil differentiation, survival, and activation, while IL-13 acts on the intestinal epithelium and vascular endothelium to promote eotaxin-3 production, resulting in eosinophil migration and accumulation in the local mucosa. IL-13 also induces periostin expression, promotes fibrosis and smooth muscle proliferation, and is involved in tissue remodeling. Transcriptome analysis of gastric and duodenal mucosal tissues from patients with non-EoE-EGID revealed an increased expression of cytokines involved in Th2-type immune responses, such as IL-4, IL-5, IL-13, and eotaxin-3 [23, 24]. Additionally, increased serum IL-5, IL-13, TARC, TSLP, and eotaxin-3 levels have been reported in patients with non-EoE-EGID [25, 26]. Therefore, the efficacy of various molecularly targeted drugs against Th2 cytokines in EGID is currently under investigation [27]. Mepolizumab is a humanized monoclonal antibody directed against interleukin-5 and is approved for the treatment of multiple type 2 inflammatory diseases, including atopic dermatitis, asthma, chronic rhinosinusitis with nasal polyps, and eosinophilic esophagitis. Two case reports showed that all three patients with non-EoE EGID who received mepolizumab administration showed clinical improvement [28, 29], with one having histologically decreased eosinophil counts. One patient showed increased eosinophil count in the mucosal tissue after mepolizumab treatment. Although mepolizumab may improve clinical symptoms in non-EoE EGID, its efficacy in suppressing eosinophilic infiltration in tissues is not yet fully clear. Future clinical trials on more patients with non-EoE EGID are needed. Dupilumab, an IL-4 receptor alpha monoclonal antibody that inhibits both IL-4 and IL-13 signaling, has been approved for the treatment of type 2 inflammatory diseases such as severe asthma and atopic dermatitis. In a clinical trial in patients with EoE aged ≥ 12 years, dupilumab significantly increased the rate of histologic remission and improved dysphagia after 24 weeks [8]; however, its efficacy in non-EoE EGID has only been reported in sporadic cases [30, 31] (Table 2). All four reported patients were children, three were males, and all four had severe atopic dermatitis or asthma. Elimination of the causative food in three cases, local steroid therapy in three cases, PPIs in three cases, and systemic steroids in two cases did not lead to remission. Dupilumab administration improved endoscopic and histologic findings after 6 weeks to 6 months, allowing one patient to discontinue systemic steroids and another to develop tolerance to the causative food. Similar to these cases, our case showed improvement in endoscopic and histologic findings 5 months after administration; systemic steroid administration was discontinued, and egg tolerance was acquired. Administration of dupilumab may lead to tolerance to the causative foods by suppressing eosinophilic inflammation in the intestine; however, this mechanism is not yet fully understood.

**Table 2:** Case reports of dupilumab administration for non-esophageal eosinophilic gastrointestinal disorders

Case	Age	Sex	Allergic Comorbidities	Endoscopic findings	Eosinophil counts (/µl)	Total IgE (IU/ml)	Previous therapy	Clinical course during the dupilumab treatment	References
1	7	M	AD (Severe), FA	Esophagitis, Duodenal ulcer, Colitis	2010	1801	Food elimination, PPI, Budesonide, Prednisolone	Improvement of endoscopic and pathological findings after 6 weeks	[27]
2	14	M	BA (Severe)	Esophagitis, Esophageal stenosis, Jejunal ulcer	1890	ND	PPI, Budesonide	Improvement of endoscopic and pathological findings after 4 months	[27]
3	9	M	AD (Severe)	Esophagitis, Gastroenteritis, Colitis	ND	ND	Food elimination, PPI, Budesonide/fluticasone, Prednisolone	Improvement of abdominal symptoms and endoscopic findings Discontinuation of budesonide/fluticasone	[27]
4	15	F	BA (Severe)	Gastroenteritis, Duodenitis Colitis	ND	ND	Milk elimination, Omalizumab, Mepolizumab	Acquisition of milk tolerance	[28]
5	15	M	AD (Severe)	Duodenal ulcer	1747	2324	Food elimination, PPI, LTRA, Prednisolone	Improvement of endoscopic and pathological findings after 5 months Discontinuation of systemic steroid administration	Present case

*AD* atopic dermatitis, *FA* food allergy, *BA* bronchial asthma, *ND* no data, *IgE* immunoglobulin E *PPI* proton pump inhibitor, *LTRA* leukotriene receptor antagonist

We also investigated the changes in the serum levels of IL-5, IL-13, and eotaxin-3 during treatment. Although no sufficient reduction of eosinophil counts was observed after the initiation of oral prednisolone, normalization of eosinophil counts was observed after the initiation of dupilumab. Similarly, serum levels of total IgE, TARC, IL-5, IL-13, and eotaxin-3 were decreased after administration of dupilumab, as expected in an atopic patient. IL-4R/IL-13R-mediated signaling suppression by dupilumab may have decreased eotaxin-3 secretion from the intestinal epithelium or vascular endothelium, as well as decreased IL-5 and IL-13 secretion from Th2 lymphocytes. Interestingly, the serum levels of IL-5 and eotaxin-3 remained within normal ranges, although the eosinophil counts in the blood increased again after discontinuation of prednisolone. A transient increase in blood eosinophil counts after the initiation of dupilumab has been reported in various atopic diseases such as bronchial asthma [32]. Given the sustained suppression of eosinophil infiltration in the gastrointestinal mucosa in our patient, dupilumab may have suppressed the extravascular migration of eosinophils into the gastrointestinal tract. However, as this is a single case, future large longitudinal cohort studies are required to validate dupilumab efficacy. A randomized clinical trial including the clinical efficacy of dupilumab and biomarker analysis in patients with non-EoE EGID is currently underway (ClinicalTrials.gov number NCT03678545), and the results are highly anticipated.

Additionally, there are no clear criteria for discontinuing dupilumab in patients with EGIDs. A reduction in esophageal eosinophil infiltration and improvements in dysphagia, weight loss, growth failure, esophageal stricture, and pathological fibrosis may be the timing of consideration for discontinuing dupilumab in patients with EoE. Similarly, for non-EoE EGID, reduction in mucosal eosinophil infiltration and improvement in abdominal pain, weight loss, and failure to thrive may be the criteria for dupilumab discontinuation. It is unclear whether long-term administration of dupilumab will lead to tolerance without EGID recurrence or side effects. This may be investigated in future research studies.

In conclusion, we presented a pediatric case of non-EoE-EGID with a duodenal ulcer. Suppression of Th2-type immune responses by dupilumab may improve abdominal symptoms as well as endoscopic and histologic findings, lead to discontinuation of systemic steroid administration, and achieve tolerance to causative foods.

### Supplementary Information

**Additional file 1: figure S1.** Details of food challenge test in this case. The test was carried out by adding one of the six types of food, which was previously eliminated, every week.

## Data Availability

All data generated or analyzed during this study are included in this published article. Further inquiries can be directed to the corresponding author.

## Abbreviations

EGID: Eosinophilic gastrointestinal disorder
EoE: Eosinophilic esophagitis
non-EoE-EGID: Non-esophageal eosinophilic gastrointestinal disorder
LTRA: Leukotriene receptor antagonist
PPI: Proton pump inhibitor
CRP: C-reactive protein
IgE: Immunoglobulin E
TARC: Thymus and activation-regulated chemokine
EASI: Eczema area and severity index
HPF: High-power field
IL: Interleukin
LST: Lymphocyte stimulation test
ILC2: Innate lymphocyte type 2
TSLP: Thymic stromal lymphopoietin
