# Supplementary figures and images for: Successful use of dupilumab for egg-induced eosinophilic gastroenteritis with duodenal ulcer: a pediatric case report and review of literature
Source: Allergy Asthma Clin Immunol. 2023 Dec 5;19:103. doi: 10.1186/s13223-023-00859-3 (PMC10698892; doi:10.1186/s13223-023-00859-3)

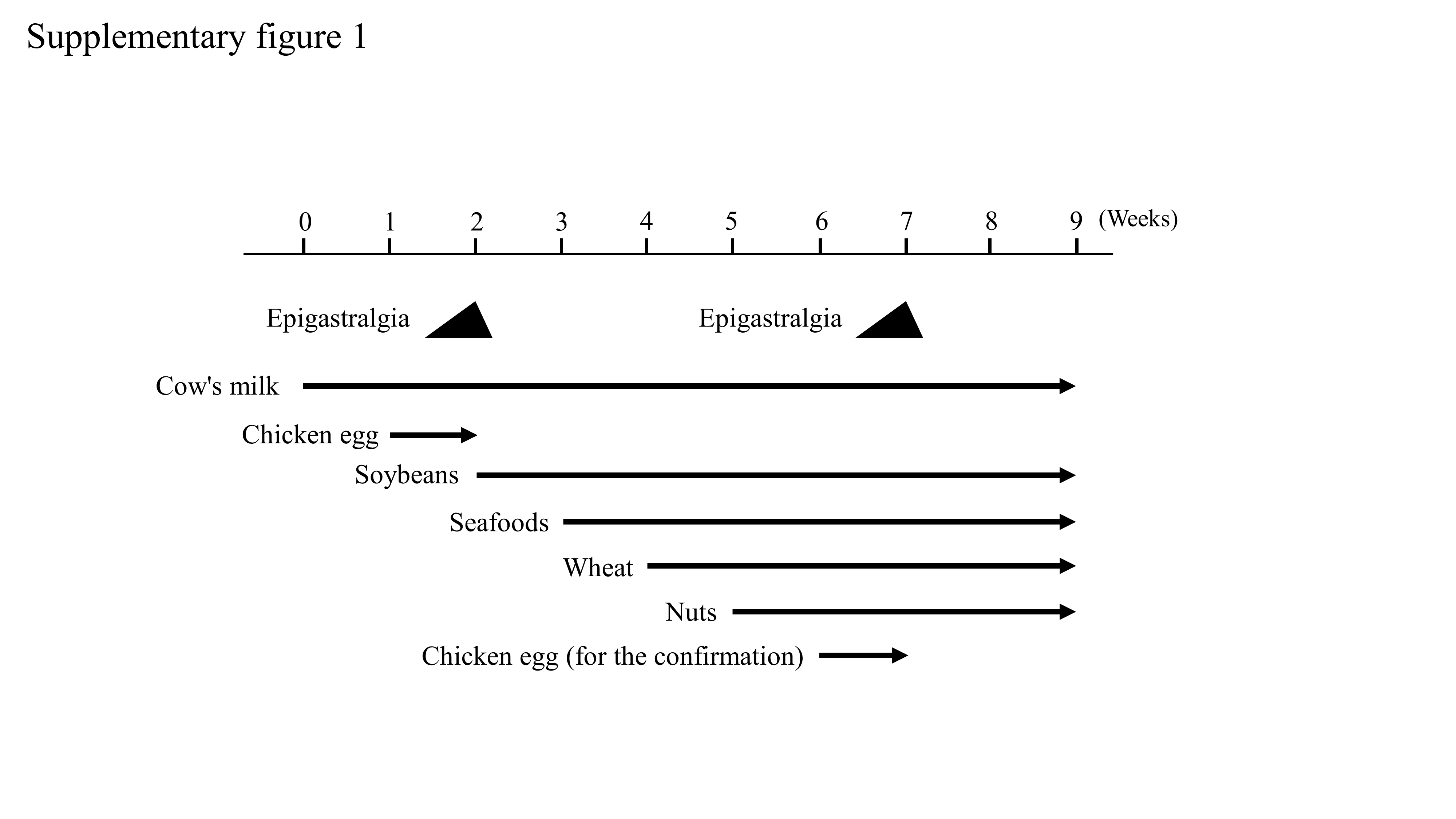

Supplement: Supplementary file 1 — Additional file 1: figure S1. Details of food challenge test in this case. The test was carried out by adding one of the six types of food, which was previously eliminated, every week. [file 13223_2023_859_MOESM1_ESM.tif]
